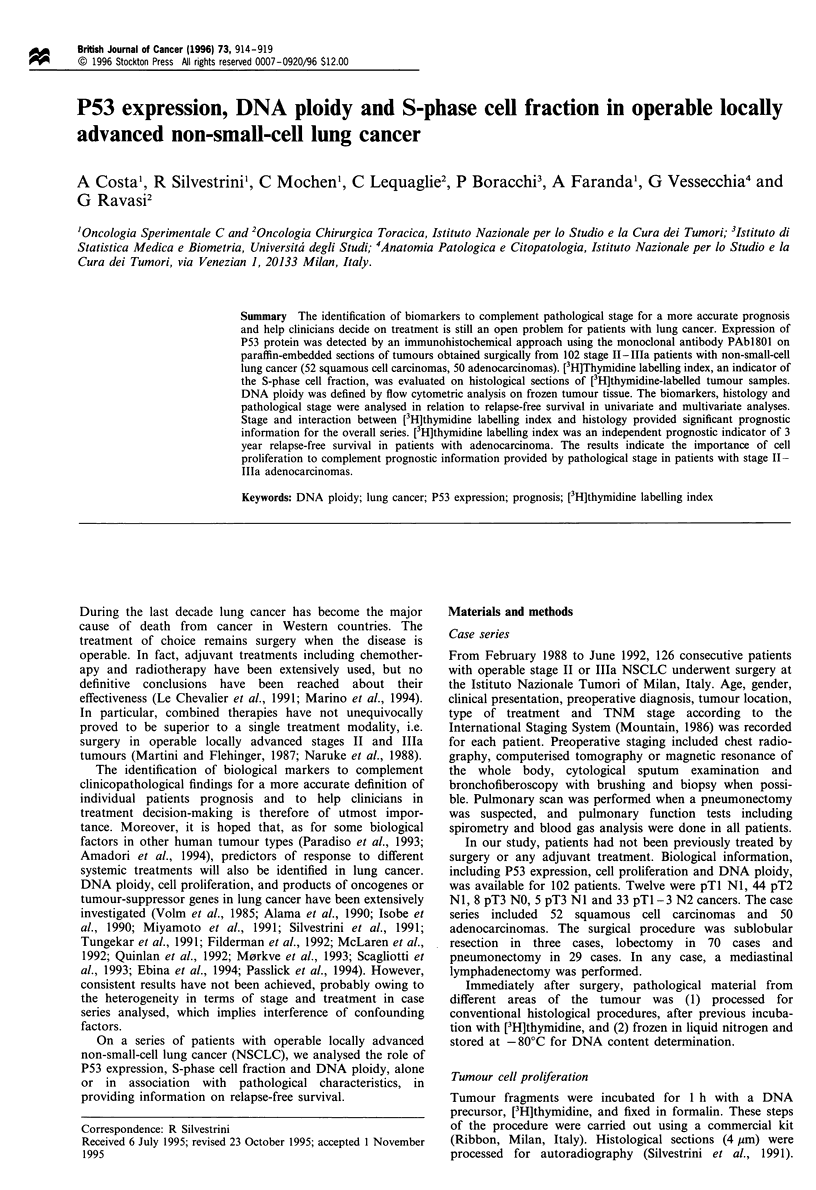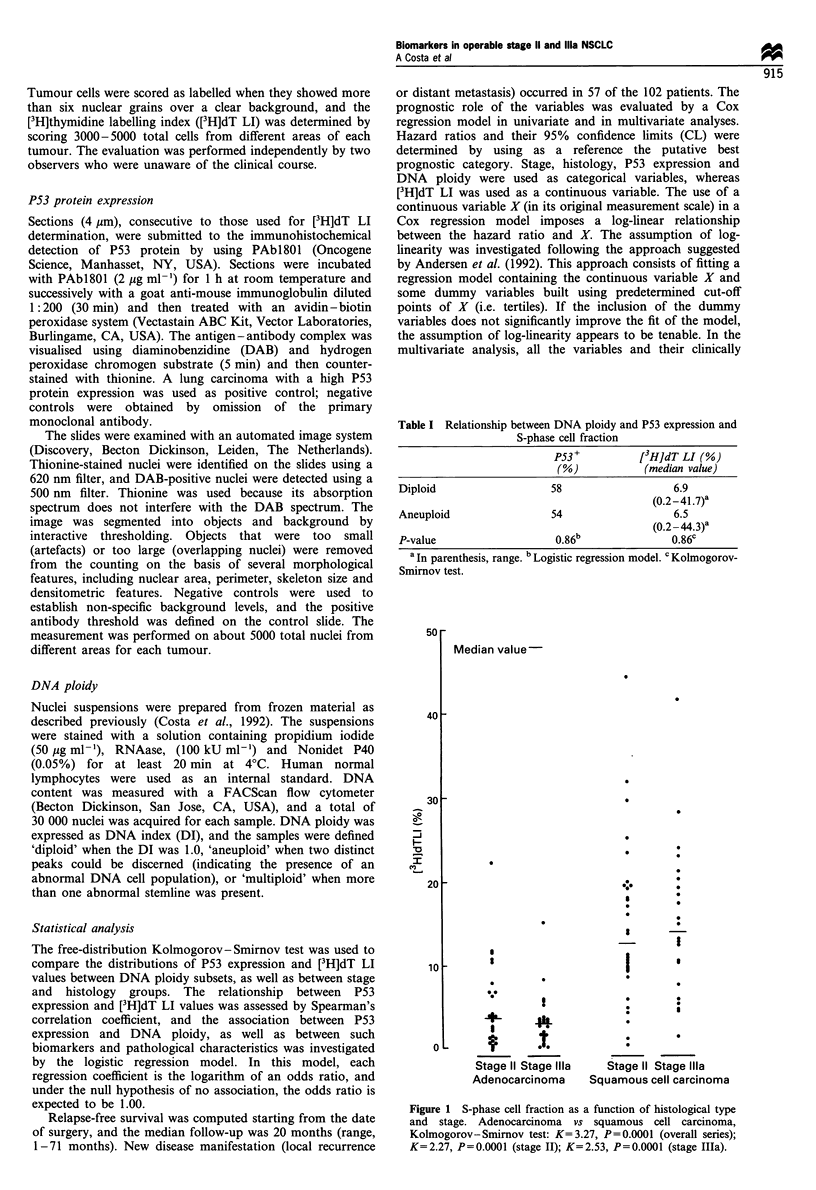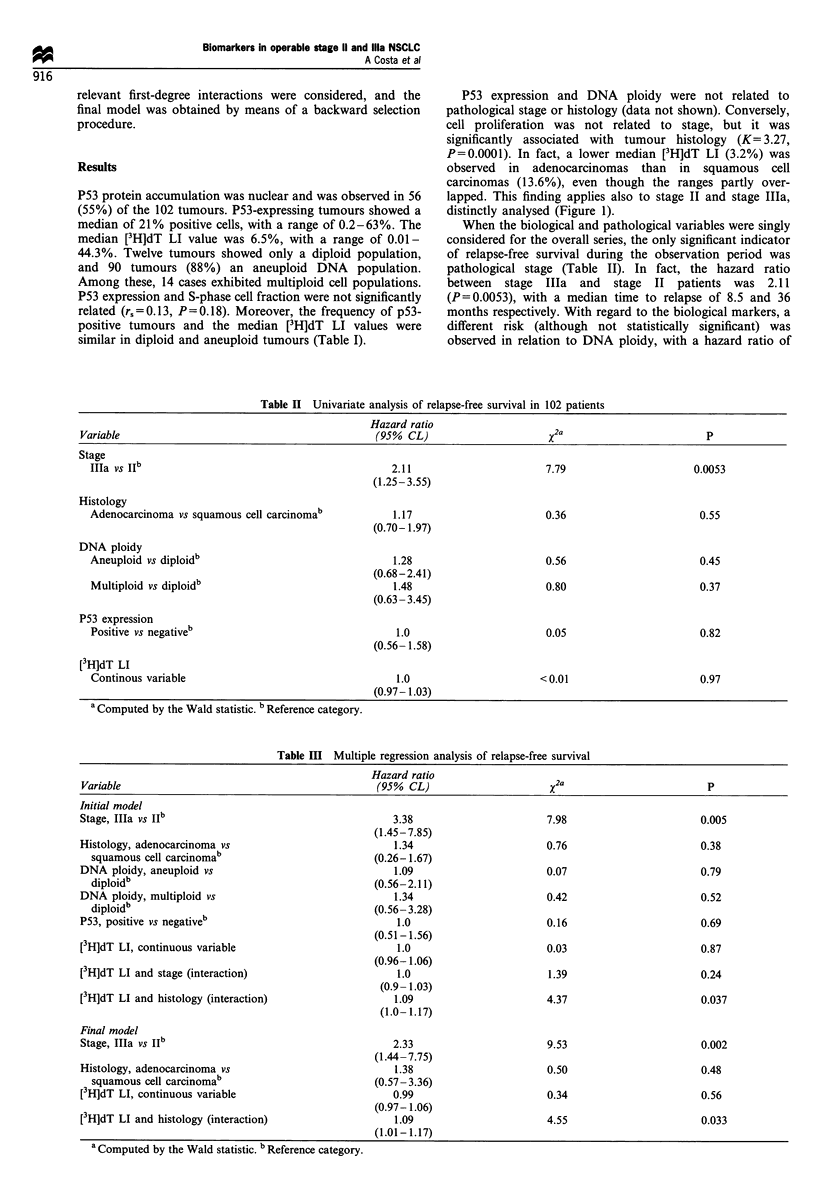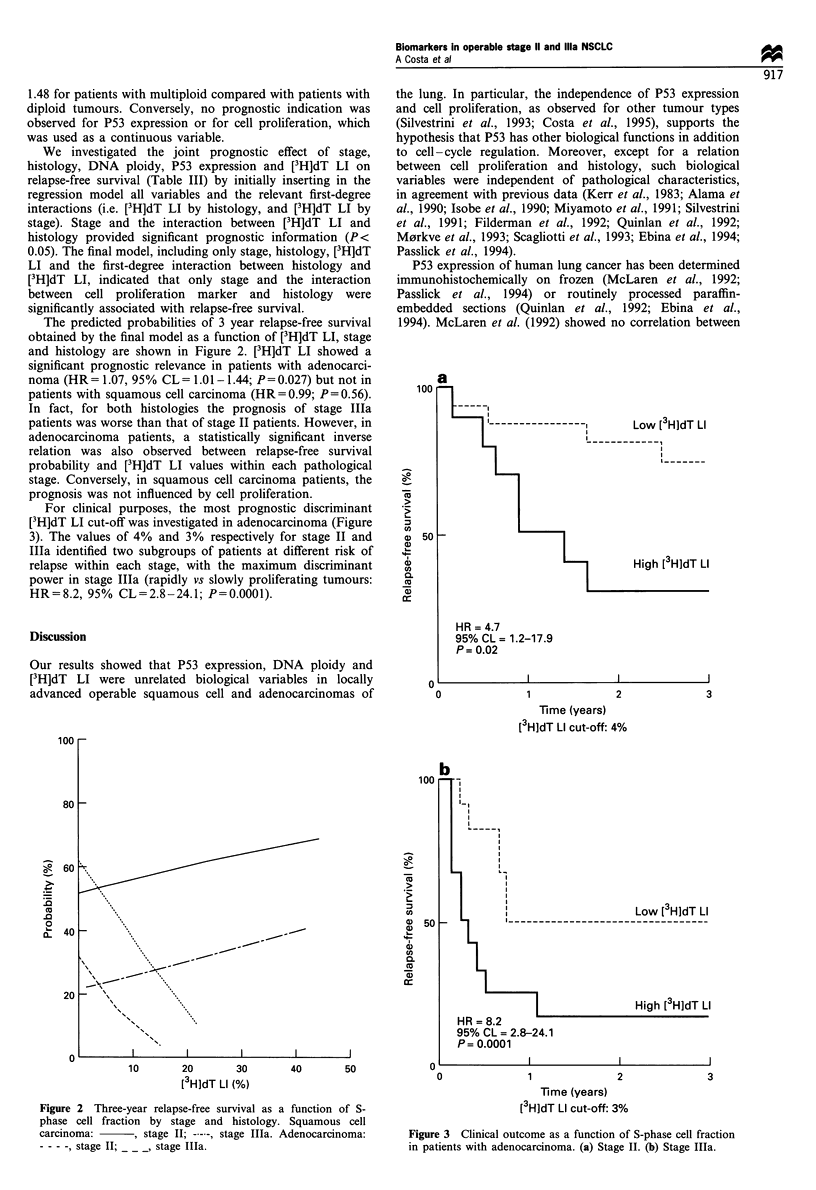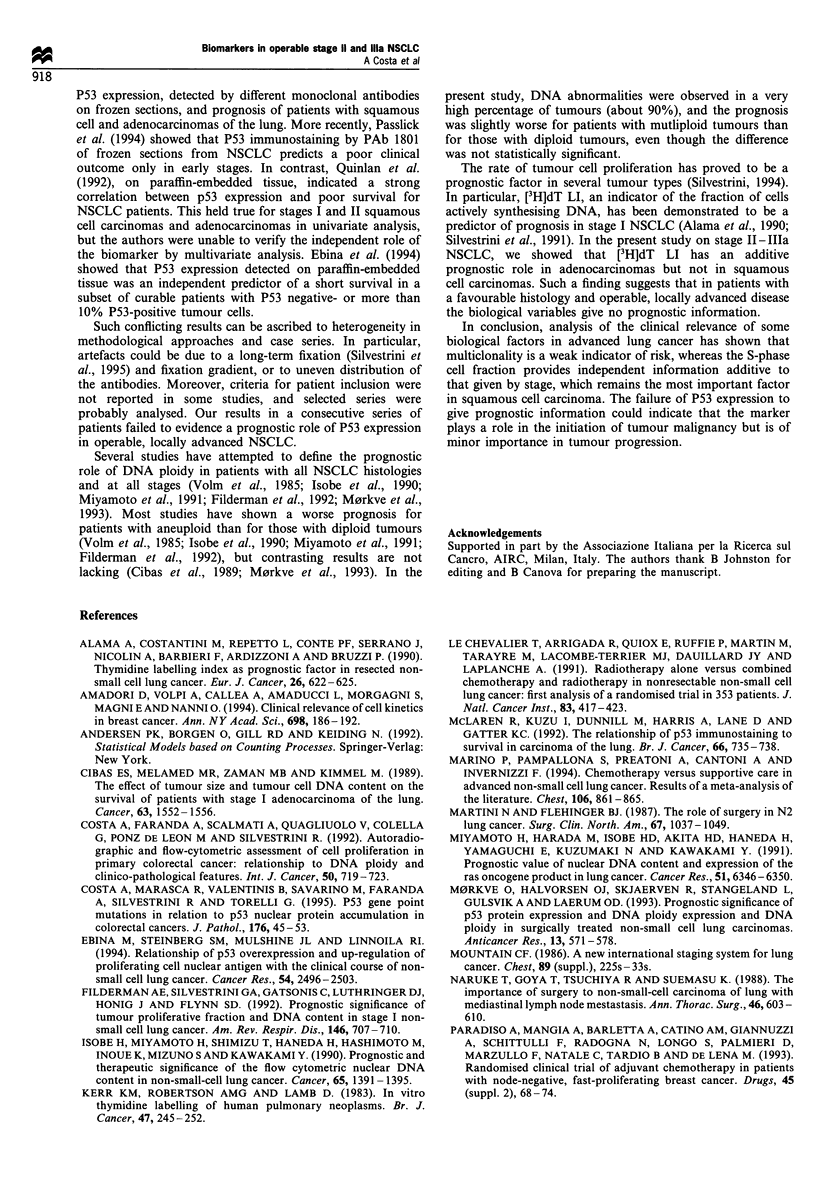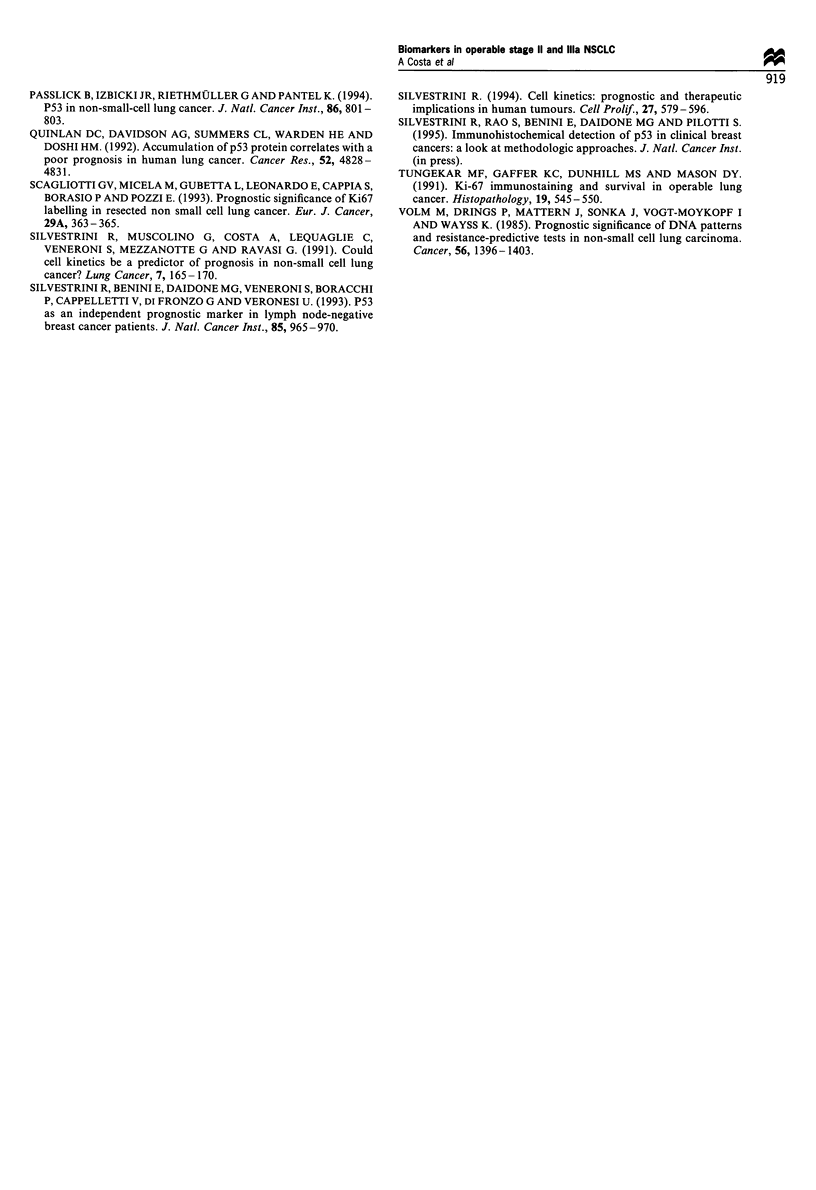# P53 expression, DNA ploidy and S-phase cell fraction in operable locally advanced non-small-cell lung cancer.

**DOI:** 10.1038/bjc.1996.163

**Published:** 1996-04

**Authors:** A. Costa, R. Silvestrini, C. Mochen, C. Lequaglie, P. Boracchi, A. Faranda, G. Vessecchia, G. Ravasi

**Affiliations:** Oncologia Sperimentale C, Istituto Nazionale per lo Studio e la Cura dei Tumori, Milan, Italy.

## Abstract

The identification of biomarkers to complement pathological stage for a more accurate prognosis and help clinicians decide on treatment is still an open problem for patients with lung cancer. Expression of P53 protein was detected by an immunohistochemical approach using the monoclonal antibody PAb1801 on paraffin-embedded sections of tumours obtained surgically from 102 stage II - IIIa patients with non-small-cell lung cancer (52 squamous cell carcinomas, 50 adenocarcinomas). [3H]Thymidine labelling index, an indicator of the S-phase cell fraction, was evaluated on histological sections of [3H]thymidine-labelled tumour samples. DNA ploidy was defined by flow cytometric analysis on frozen tumour tissue. The biomarkers, histology and pathological stage were analysed in relation to relapse-free survival in univariate and multivariate analyses. Stage and interaction between [3H]thymidine labelling index and histology provided significant prognostic information for the overall series. [3H]thymidine labelling index was an independent prognostic indicator of 3 year relapse-free survival in patients with adenocarcinoma. The results indicate the importance of cell proliferation to complement prognostic information provided by pathological stage in patients with stage II-IIIa adenocarcinomas.


British Journal of Cancer (1996) 73, 914-919
© 1996 Stockton Press All rights reserved 0007-0920/96 $12.00

### P53 expression, DNA ploidy and S-phase cell fraction in operable locally advanced non-small-cell lung cancer

A Costa[1], R Silvestrini[1], C Mochen[1], C Lequaglie[2], P Boracchi[3], A Faranda[1], G Vessecchia[4] and G Ravasi[2]

*[1]Oncologia Sperimentale C and [2]Oncologia Chirurgica Toracica, Istituto Nazionale per lo Studio e la Cura dei Tumori; [3]Istituto di Statistica Medica e Biometria, Universitá degli Studi; [4]Anatomia Patologica e Citopatologia, Istituto Nazionale per lo Studio e la Cura dei Tumori, via Venezian 1, 20133 Milan, Italy.*

**Summary** The identification of biomarkers to complement pathological stage for a more accurate prognosis and help clinicians decide on treatment is still an open problem for patients with lung cancer. Expression of P53 protein was detected by an immunohistochemical approach using the monoclonal antibody PAb1801 on paraffin-embedded sections of tumours obtained surgically from 102 stage II–IIIa patients with non-small-cell lung cancer (52 squamous cell carcinomas, 50 adenocarcinomas). [$^3$H]Thymidine labelling index, an indicator of the S-phase cell fraction, was evaluated on histological sections of [$^3$H]thymidine-labelled tumour samples. DNA ploidy was defined by flow cytometric analysis on frozen tumour tissue. The biomarkers, histology and pathological stage were analysed in relation to relapse-free survival in univariate and multivariate analyses. Stage and interaction between [$^3$H]thymidine labelling index and histology provided significant prognostic information for the overall series. [$^3$H]thymidine labelling index was an independent prognostic indicator of 3 year relapse-free survival in patients with adenocarcinoma. The results indicate the importance of cell proliferation to complement prognostic information provided by pathological stage in patients with stage II–IIIa adenocarcinomas.

**Keywords:** DNA ploidy; lung cancer; P53 expression; prognosis; [$^3$H]thymidine labelling index

During the last decade lung cancer has become the major cause of death from cancer in Western countries. The treatment of choice remains surgery when the disease is operable. In fact, adjuvant treatments including chemotherapy and radiotherapy have been extensively used, but no definitive conclusions have been reached about their effectiveness (Le Chevalier *et al.*, 1991; Marino *et al.*, 1994). In particular, combined therapies have not unequivocally proved to be superior to a single treatment modality, i.e. surgery in operable locally advanced stages II and IIIa tumours (Martini and Flehinger, 1987; Naruke *et al.*, 1988).

The identification of biological markers to complement clinicopathological findings for a more accurate definition of individual patients prognosis and to help clinicians in treatment decision-making is therefore of utmost importance. Moreover, it is hoped that, as for some biological factors in other human tumour types (Paradiso *et al.*, 1993; Amadori *et al.*, 1994), predictors of response to different systemic treatments will also be identified in lung cancer. DNA ploidy, cell proliferation, and products of oncogenes or tumour-suppressor genes in lung cancer have been extensively investigated (Volm *et al.*, 1985; Alama *et al.*, 1990; Isobe *et al.*, 1990; Miyamoto *et al.*, 1991; Silvestrini *et al.*, 1991; Tungekar *et al.*, 1991; Filderman *et al.*, 1992; McLaren *et al.*, 1992; Quinlan *et al.*, 1992; Mørkve *et al.*, 1993; Scagliotti *et al.*, 1993; Ebina *et al.*, 1994; Passlick *et al.*, 1994). However, consistent results have not been achieved, probably owing to the heterogeneity in terms of stage and treatment in case series analysed, which implies interference of confounding factors.

On a series of patients with operable locally advanced non-small-cell lung cancer (NSCLC), we analysed the role of P53 expression, S-phase cell fraction and DNA ploidy, alone or in association with pathological characteristics, in providing information on relapse-free survival.

Correspondence: R Silvestrini
Received 6 July 1995; revised 23 October 1995; accepted 1 November 1995

### Materials and methods

### *Case series*

From February 1988 to June 1992, 126 consecutive patients with operable stage II or IIIa NSCLC underwent surgery at the Istituto Nazionale Tumori of Milan, Italy. Age, gender, clinical presentation, preoperative diagnosis, tumour location, type of treatment and TNM stage according to the International Staging System (Mountain, 1986) was recorded for each patient. Preoperative staging included chest radiography, computerised tomography or magnetic resonance of the whole body, cytological sputum examination and bronchofiberoscopy with brushing and biopsy when possible. Pulmonary scan was performed when a pneumonectomy was suspected, and pulmonary function tests including spirometry and blood gas analysis were done in all patients.

In our study, patients had not been previously treated by surgery or any adjuvant treatment. Biological information, including P53 expression, cell proliferation and DNA ploidy, was available for 102 patients. Twelve were pT1 N1, 44 pT2 N1, 8 pT3 N0, 5 pT3 N1 and 33 pT1–3 N2 cancers. The case series included 52 squamous cell carcinomas and 50 adenocarcinomas. The surgical procedure was sublobular resection in three cases, lobectomy in 70 cases and pneumonectomy in 29 cases. In any case, a mediastinal lymphadenectomy was performed.

Immediately after surgery, pathological material from different areas of the tumour was (1) processed for conventional histological procedures, after previous incubation with [$^3$H]thymidine, and (2) frozen in liquid nitrogen and stored at $-80$°C for DNA content determination.

### *Tumour cell proliferation*

Tumour fragments were incubated for 1 h with a DNA precursor, [$^3$H]thymidine, and fixed in formalin. These steps of the procedure were carried out using a commercial kit (Ribbon, Milan, Italy). Histological sections (4 $\mu$m) were processed for autoradiography (Silvestrini *et al.*, 1991).

Tumour cells were scored as labelled when they showed more than six nuclear grains over a clear background, and the [$^3$H]thymidine labelling index ([$^3$H]dT LI) was determined by scoring 3000–5000 total cells from different areas of each tumour. The evaluation was performed independently by two observers who were unaware of the clinical course.

*P53 protein expression*

Sections (4 $\mu$m), consecutive to those used for [$^3$H]dT LI determination, were submitted to the immunohistochemical detection of P53 protein by using PAb1801 (Oncogene Science, Manhasset, NY, USA). Sections were incubated with PAb1801 (2 $\mu$g ml$^{-1}$) for 1 h at room temperature and successively with a goat anti-mouse immunoglobulin diluted 1:200 (30 min) and then treated with an avidin–biotin peroxidase system (Vectastain ABC Kit, Vector Laboratories, Burlingame, CA, USA). The antigen–antibody complex was visualised using diaminobenzidine (DAB) and hydrogen peroxidase chromogen substrate (5 min) and then counter-stained with thionine. A lung carcinoma with a high P53 protein expression was used as positive control; negative controls were obtained by omission of the primary monoclonal antibody.

The slides were examined with an automated image system (Discovery, Becton Dickinson, Leiden, The Netherlands). Thionine-stained nuclei were identified on the slides using a 620 nm filter, and DAB-positive nuclei were detected using a 500 nm filter. Thionine was used because its absorption spectrum does not interfere with the DAB spectrum. The image was segmented into objects and background by interactive thresholding. Objects that were too small (artefacts) or too large (overlapping nuclei) were removed from the counting on the basis of several morphological features, including nuclear area, perimeter, skeleton size and densitometric features. Negative controls were used to establish non-specific background levels, and the positive antibody threshold was defined on the control slide. The measurement was performed on about 5000 total nuclei from different areas for each tumour.

*DNA ploidy*

Nuclei suspensions were prepared from frozen material as described previously (Costa *et al.*, 1992). The suspensions were stained with a solution containing propidium iodide (50 $\mu$g ml$^{-1}$), RNAase, (100 kU ml$^{-1}$) and Nonidet P40 (0.05%) for at least 20 min at 4°C. Human normal lymphocytes were used as an internal standard. DNA content was measured with a FACScan flow cytometer (Becton Dickinson, San Jose, CA, USA), and a total of 30 000 nuclei was acquired for each sample. DNA ploidy was expressed as DNA index (DI), and the samples were defined 'diploid' when the DI was 1.0, 'aneuploid' when two distinct peaks could be discerned (indicating the presence of an abnormal DNA cell population), or 'multiploid' when more than one abnormal stemline was present.

*Statistical analysis*

The free-distribution Kolmogorov–Smirnov test was used to compare the distributions of P53 expression and [$^3$H]dT LI values between DNA ploidy subsets, as well as between stage and histology groups. The relationship between P53 expression and [$^3$H]dT LI values was assessed by Spearman's correlation coefficient, and the association between P53 expression and DNA ploidy, as well as between such biomarkers and pathological characteristics was investigated by the logistic regression model. In this model, each regression coefficient is the logarithm of an odds ratio, and under the null hypothesis of no association, the odds ratio is expected to be 1.00.

Relapse-free survival was computed starting from the date of surgery, and the median follow-up was 20 months (range, 1–71 months). New disease manifestation (local recurrence or distant metastasis) occurred in 57 of the 102 patients. The prognostic role of the variables was evaluated by a Cox regression model in univariate and in multivariate analyses. Hazard ratios and their 95% confidence limits (CL) were determined by using as a reference the putative best prognostic category. Stage, histology, P53 expression and DNA ploidy were used as categorical variables, whereas [$^3$H]dT LI was used as a continuous variable. The use of a continuous variable $X$ (in its original measurement scale) in a Cox regression model imposes a log-linear relationship between the hazard ratio and $X$. The assumption of log-linearity was investigated following the approach suggested by Andersen *et al.* (1992). This approach consists of fitting a regression model containing the continuous variable $X$ and some dummy variables built using predetermined cut-off points of $X$ (i.e. tertiles). If the inclusion of the dummy variables does not significantly improve the fit of the model, the assumption of log-linearity appears to be tenable. In the multivariate analysis, all the variables and their clinically

**Table I** Relationship between DNA ploidy and P53 expression and S-phase cell fraction

| | *P53$^+$ (%)* | *[$^3$H]dT LI (%) (median value)* |
|---|---|---|
| Diploid | 58 | 6.9 (0.2–41.7)[a] |
| Aneuploid | 54 | 6.5 (0.2–44.3)[a] |
| *P*-value | 0.86[b] | 0.86[c] |

[a] In parenthesis, range. [b] Logistic regression model. [c] Kolmogorov-Smirnov test.

**Figure 1** S-phase cell fraction as a function of histological type and stage. Adenocarcinoma *vs* squamous cell carcinoma, Kolmogorov–Smirnov test: $K$=3.27, $P$=0.0001 (overall series); $K$=2.27, $P$=0.0001 (stage II); $K$=2.53, $P$=0.0001 (stage IIIa).

relevant first-degree interactions were considered, and the final model was obtained by means of a backward selection procedure.

### Results

P53 protein accumulation was nuclear and was observed in 56 (55%) of the 102 tumours. P53-expressing tumours showed a median of 21% positive cells, with a range of 0.2–63%. The median [$^3$H]dT LI value was 6.5%, with a range of 0.01–44.3%. Twelve tumours showed only a diploid population, and 90 tumours (88%) an aneuploid DNA population. Among these, 14 cases exhibited multiploid cell populations. P53 expression and S-phase cell fraction were not significantly related ($r_s$=0.13, $P$=0.18). Moreover, the frequency of p53-positive tumours and the median [$^3$H]dT LI values were similar in diploid and aneuploid tumours (Table I).

P53 expression and DNA ploidy were not related to pathological stage or histology (data not shown). Conversely, cell proliferation was not related to stage, but it was significantly associated with tumour histology ($K$=3.27, $P$=0.0001). In fact, a lower median [$^3$H]dT LI (3.2%) was observed in adenocarcinomas than in squamous cell carcinomas (13.6%), even though the ranges partly overlapped. This finding applies also to stage II and stage IIIa, distinctly analysed (Figure 1).

When the biological and pathological variables were singly considered for the overall series, the only significant indicator of relapse-free survival during the observation period was pathological stage (Table II). In fact, the hazard ratio between stage IIIa and stage II patients was 2.11 ($P$=0.0053), with a median time to relapse of 8.5 and 36 months respectively. With regard to the biological markers, a different risk (although not statistically significant) was observed in relation to DNA ploidy, with a hazard ratio of

**Table II** Univariate analysis of relapse-free survival in 102 patients

| *Variable* | *Hazard ratio (95% CL)* | $\chi^{2a}$ | P |
|---|---|---|---|
| Stage | | | |
| IIIa *vs* II[b] | 2.11 (1.25–3.55) | 7.79 | 0.0053 |
| Histology | | | |
| Adenocarcinoma *vs* squamous cell carcinoma[b] | 1.17 (0.70–1.97) | 0.36 | 0.55 |
| DNA ploidy | | | |
| Aneuploid *vs* diploid[b] | 1.28 (0.68–2.41) | 0.56 | 0.45 |
| Multiploid *vs* diploid[b] | 1.48 (0.63–3.45) | 0.80 | 0.37 |
| P53 expression | | | |
| Positive *vs* negative[b] | 1.0 (0.56–1.58) | 0.05 | 0.82 |
| [$^3$H]dT LI | | | |
| Continous variable | 1.0 (0.97–1.03) | <0.01 | 0.97 |

[a] Computed by the Wald statistic. [b] Reference category.

**Table III** Multiple regression analysis of relapse-free survival

| *Variable* | *Hazard ratio (95% CL)* | $\chi^{2a}$ | P |
|---|---|---|---|
| *Initial model* | | | |
| Stage, IIIa *vs* II[b] | 3.38 (1.45–7.85) | 7.98 | 0.005 |
| Histology, adenocarcinoma *vs* squamous cell carcinoma[b] | 1.34 (0.26–1.67) | 0.76 | 0.38 |
| DNA ploidy, aneuploid *vs* diploid[b] | 1.09 (0.56–2.11) | 0.07 | 0.79 |
| DNA ploidy, multiploid *vs* diploid[b] | 1.34 (0.56–3.28) | 0.42 | 0.52 |
| P53, positive *vs* negative[b] | 1.0 (0.51–1.56) | 0.16 | 0.69 |
| [$^3$H]dT LI, continuous variable | 1.0 (0.96–1.06) | 0.03 | 0.87 |
| [$^3$H]dT LI and stage (interaction) | 1.0 (0.9–1.03) | 1.39 | 0.24 |
| [$^3$H]dT LI and histology (interaction) | 1.09 (1.0–1.17) | 4.37 | 0.037 |
| *Final model* | | | |
| Stage, IIIa *vs* II[b] | 2.33 (1.44–7.75) | 9.53 | 0.002 |
| Histology, adenocarcinoma *vs* squamous cell carcinoma[b] | 1.38 (0.57–3.36) | 0.50 | 0.48 |
| [$^3$H]dT LI, continuous variable | 0.99 (0.97–1.06) | 0.34 | 0.56 |
| [$^3$H]dT LI and histology (interaction) | 1.09 (1.01–1.17) | 4.55 | 0.033 |

[a] Computed by the Wald statistic. [b] Reference category.

1.48 for patients with multiploid compared with patients with diploid tumours. Conversely, no prognostic indication was observed for P53 expression or for cell proliferation, which was used as a continuous variable.

We investigated the joint prognostic effect of stage, histology, DNA ploidy, P53 expression and [$^3$H]dT LI on relapse-free survival (Table III) by initially inserting in the regression model all variables and the relevant first-degree interactions (i.e. [$^3$H]dT LI by histology, and [$^3$H]dT LI by stage). Stage and the interaction between [$^3$H]dT LI and histology provided significant prognostic information ($P<0.05$). The final model, including only stage, histology, [$^3$H]dT LI and the first-degree interaction between histology and [$^3$H]dT LI, indicated that only stage and the interaction between cell proliferation marker and histology were significantly associated with relapse-free survival.

The predicted probabilities of 3 year relapse-free survival obtained by the final model as a function of [$^3$H]dT LI, stage and histology are shown in Figure 2. [$^3$H]dT LI showed a significant prognostic relevance in patients with adenocarcinoma (HR = 1.07, 95% CL = 1.01–1.44; $P=0.027$) but not in patients with squamous cell carcinoma (HR = 0.99; $P=0.56$). In fact, for both histologies the prognosis of stage IIIa patients was worse than that of stage II patients. However, in adenocarcinoma patients, a statistically significant inverse relation was also observed between relapse-free survival probability and [$^3$H]dT LI values within each pathological stage. Conversely, in squamous cell carcinoma patients, the prognosis was not influenced by cell proliferation.

For clinical purposes, the most prognostic discriminant [$^3$H]dT LI cut-off was investigated in adenocarcinoma (Figure 3). The values of 4% and 3% respectively for stage II and IIIa identified two subgroups of patients at different risk of relapse within each stage, with the maximum discriminant power in stage IIIa (rapidly *vs* slowly proliferating tumours: HR = 8.2, 95% CL = 2.8–24.1; $P=0.0001$).

### Discussion

Our results showed that P53 expression, DNA ploidy and [$^3$H]dT LI were unrelated biological variables in locally advanced operable squamous cell and adenocarcinomas of the lung. In particular, the independence of P53 expression and cell proliferation, as observed for other tumour types (Silvestrini *et al.*, 1993; Costa *et al.*, 1995), supports the hypothesis that P53 has other biological functions in addition to cell–cycle regulation. Moreover, except for a relation between cell proliferation and histology, such biological variables were independent of pathological characteristics, in agreement with previous data (Kerr *et al.*, 1983; Alama *et al.*, 1990; Isobe *et al.*, 1990; Miyamoto *et al.*, 1991; Silvestrini *et al.*, 1991; Filderman *et al.*, 1992; Quinlan *et al.*, 1992; Mørkve *et al.*, 1993; Scagliotti *et al.*, 1993; Ebina *et al.*, 1994; Passlick *et al.*, 1994).

P53 expression of human lung cancer has been determined immunohistochemically on frozen (McLaren *et al.*, 1992; Passlick *et al.*, 1994) or routinely processed paraffin-embedded sections (Quinlan *et al.*, 1992; Ebina *et al.*, 1994). McLaren *et al.* (1992) showed no correlation between

**Figure 2** Three-year relapse-free survival as a function of S-phase cell fraction by stage and histology. Squamous cell carcinoma: ———, stage II; -·-·-, stage IIIa. Adenocarcinoma: - - - -, stage II; _ _ _, stage IIIa.

**Figure 3** Clinical outcome as a function of S-phase cell fraction in patients with adenocarcinoma. (**a**) Stage II. (**b**) Stage IIIa.

P53 expression, detected by different monoclonal antibodies on frozen sections, and prognosis of patients with squamous cell and adenocarcinomas of the lung. More recently, Passlick *et al.* (1994) showed that P53 immunostaining by PAb 1801 of frozen sections from NSCLC predicts a poor clinical outcome only in early stages. In contrast, Quinlan *et al.* (1992), on paraffin-embedded tissue, indicated a strong correlation between p53 expression and poor survival for NSCLC patients. This held true for stages I and II squamous cell carcinomas and adenocarcinomas in univariate analysis, but the authors were unable to verify the independent role of the biomarker by multivariate analysis. Ebina *et al.* (1994) showed that P53 expression detected on paraffin-embedded tissue was an independent predictor of a short survival in a subset of curable patients with P53 negative- or more than 10% P53-positive tumour cells.

Such conflicting results can be ascribed to heterogeneity in methodological approaches and case series. In particular, artefacts could be due to a long-term fixation (Silvestrini *et al.*, 1995) and fixation gradient, or to uneven distribution of the antibodies. Moreover, criteria for patient inclusion were not reported in some studies, and selected series were probably analysed. Our results in a consecutive series of patients failed to evidence a prognostic role of P53 expression in operable, locally advanced NSCLC.

Several studies have attempted to define the prognostic role of DNA ploidy in patients with all NSCLC histologies and at all stages (Volm *et al.*, 1985; Isobe *et al.*, 1990; Miyamoto *et al.*, 1991; Filderman *et al.*, 1992; Mørkve *et al.*, 1993). Most studies have shown a worse prognosis for patients with aneuploid than for those with diploid tumours (Volm *et al.*, 1985; Isobe *et al.*, 1990; Miyamoto *et al.*, 1991; Filderman *et al.*, 1992), but contrasting results are not lacking (Cibas *et al.*, 1989; Mørkve *et al.*, 1993). In the present study, DNA abnormalities were observed in a very high percentage of tumours (about 90%), and the prognosis was slightly worse for patients with mutliploid tumours than for those with diploid tumours, even though the difference was not statistically significant.

The rate of tumour cell proliferation has proved to be a prognostic factor in several tumour types (Silvestrini, 1994). In particular, [$^3$H]dT LI, an indicator of the fraction of cells actively synthesising DNA, has been demonstrated to be a predictor of prognosis in stage I NSCLC (Alama *et al.*, 1990; Silvestrini *et al.*, 1991). In the present study on stage II–IIIa NSCLC, we showed that [$^3$H]dT LI has an additive prognostic role in adenocarcinomas but not in squamous cell carcinomas. Such a finding suggests that in patients with a favourable histology and operable, locally advanced disease the biological variables give no prognostic information.

In conclusion, analysis of the clinical relevance of some biological factors in advanced lung cancer has shown that multiclonality is a weak indicator of risk, whereas the S-phase cell fraction provides independent information additive to that given by stage, which remains the most important factor in squamous cell carcinoma. The failure of P53 expression to give prognostic information could indicate that the marker plays a role in the initiation of tumour malignancy but is of minor importance in tumour progression.

**Acknowledgements**
Supported in part by the Associazione Italiana per la Ricerca sul Cancro, AIRC, Milan, Italy. The authors thank B Johnston for editing and B Canova for preparing the manuscript.

### References

ALAMA A, COSTANTINI M, REPETTO L, CONTE PF, SERRANO J, NICOLIN A, BARBIERI F, ARDIZZONI A AND BRUZZI P. (1990). Thymidine labelling index as prognostic factor in resected non-small cell lung cancer. *Eur. J. Cancer*, **26**, 622–625.

AMADORI D, VOLPI A, CALLEA A, AMADUCCI L, MORGAGNI S, MAGNI E AND NANNI O. (1994). Clinical relevance of cell kinetics in breast cancer. *Ann. NY Acad. Sci.*, **698**, 186–192.

ANDERSEN PK, BORGEN O, GILL RD AND KEIDING N. (1992). *Statistical Models based on Counting Processes*. Springer-Verlag: New York.

CIBAS ES, MELAMED MR, ZAMAN MB AND KIMMEL M. (1989). The effect of tumour size and tumour cell DNA content on the survival of patients with stage I adenocarcinoma of the lung. *Cancer*, **63**, 1552–1556.

COSTA A, FARANDA A, SCALMATI A, QUAGLIUOLO V, COLELLA G, PONZ DE LEON M AND SILVESTRINI R. (1992). Autoradiographic and flow-cytometric assessment of cell proliferation in primary colorectal cancer: relationship to DNA ploidy and clinico-pathological features. *Int. J. Cancer*, **50**, 719–723.

COSTA A, MARASCA R, VALENTINIS B, SAVARINO M, FARANDA A, SILVESTRINI R AND TORELLI G. (1995). P53 gene point mutations in relation to p53 nuclear protein accumulation in colorectal cancers. *J. Pathol.*, **176**, 45–53.

EBINA M, STEINBERG SM, MULSHINE JL AND LINNOILA RI. (1994). Relationship of p53 overexpression and up-regulation of proliferating cell nuclear antigen with the clinical course of non-small cell lung cancer. *Cancer Res.*, **54**, 2496–2503.

FILDERMAN AE, SILVESTRINI GA, GATSONIS C, LUTHRINGER DJ, HONIG J AND FLYNN SD. (1992). Prognostic significance of tumour proliferative fraction and DNA content in stage I non-small cell lung cancer. *Am. Rev. Respir. Dis.*, **146**, 707–710.

ISOBE H, MIYAMOTO H, SHIMIZU T, HANEDA H, HASHIMOTO M, INOUE K, MIZUNO S AND KAWAKAMI Y. (1990). Prognostic and therapeutic significance of the flow cytometric nuclear DNA content in non-small-cell lung cancer. *Cancer*, **65**, 1391–1395.

KERR KM, ROBERTSON AMG AND LAMB D. (1983). In vitro thymidine labelling of human pulmonary neoplasms. *Br. J. Cancer*, **47**, 245–252.

LE CHEVALIER T, ARRIGADA R, QUIOX E, RUFFIE P, MARTIN M, TARAYRE M, LACOMBE-TERRIER MJ, DAUILLARD JY AND LAPLANCHE A. (1991). Radiotherapy alone versus combined chemotherapy and radiotherapy in nonresectable non-small cell lung cancer: first analysis of a randomised trial in 353 patients. *J. Natl. Cancer Inst.*, **83**, 417–423.

MCLAREN R, KUZU I, DUNNILL M, HARRIS A, LANE D AND GATTER KC. (1992). The relationship of p53 immunostaining to survival in carcinoma of the lung. *Br. J. Cancer*, **66**, 735–738.

MARINO P, PAMPALLONA S, PREATONI A, CANTONI A AND INVERNIZZI F. (1994). Chemotherapy versus supportive care in advanced non-small cell lung cancer. Results of a meta-analysis of the literature. *Chest*, **106**, 861–865.

MARTINI N AND FLEHINGER BJ. (1987). The role of surgery in N2 lung cancer. *Surg. Clin. North. Am.*, **67**, 1037–1049.

MIYAMOTO H, HARADA M, ISOBE HD, AKITA HD, HANEDA H, YAMAGUCHI E, KUZUMAKI N AND KAWAKAMI Y. (1991). Prognostic value of nuclear DNA content and expression of the ras oncogene product in lung cancer. *Cancer Res.*, **51**, 6346–6350.

MØRKVE O, HALVORSEN OJ, SKJAERVEN R, STANGELAND L, GULSVIK A AND LAERUM OD. (1993). Prognostic significance of p53 protein expression and DNA ploidy expression and DNA ploidy in surgically treated non-small cell lung carcinomas. *Anticancer Res.*, **13**, 571–578.

MOUNTAIN CF. (1986). A new international staging system for lung cancer. *Chest*, **89** (suppl.), 225s–33s.

NARUKE T, GOYA T, TSUCHIYA R AND SUEMASU K. (1988). The importance of surgery to non-small-cell carcinoma of lung with mediastinal lymph node mestastasis. *Ann. Thorac. Surg.*, **46**, 603–610.

PARADISO A, MANGIA A, BARLETTA A, CATINO AM, GIANNUZZI A, SCHITTULLI F, RADOGNA N, LONGO S, PALMIERI D, MARZULLO F, NATALE C, TARDIO B AND DE LENA M. (1993). Randomised clinical trial of adjuvant chemotherapy in patients with node-negative, fast-proliferating breast cancer. *Drugs*, **45** (suppl. 2), 68–74.

PASSLICK B, IZBICKI JR, RIETHMÜLLER G AND PANTEL K. (1994). P53 in non-small-cell lung cancer. *J. Natl. Cancer Inst.*, **86,** 801–803.

QUINLAN DC, DAVIDSON AG, SUMMERS CL, WARDEN HE AND DOSHI HM. (1992). Accumulation of p53 protein correlates with a poor prognosis in human lung cancer. *Cancer Res.*, **52,** 4828–4831.

SCAGLIOTTI GV, MICELA M, GUBETTA L, LEONARDO E, CAPPIA S, BORASIO P AND POZZI E. (1993). Prognostic significance of Ki67 labelling in resected non small cell lung cancer. *Eur. J. Cancer*, **29A,** 363–365.

SILVESTRINI R, MUSCOLINO G, COSTA A, LEQUAGLIE C, VENERONI S, MEZZANOTTE G AND RAVASI G. (1991). Could cell kinetics be a predictor of prognosis in non-small cell lung cancer? *Lung Cancer*, **7,** 165–170.

SILVESTRINI R, BENINI E, DAIDONE MG, VENERONI S, BORACCHI P, CAPPELLETTI V, DI FRONZO G AND VERONESI U. (1993). P53 as an independent prognostic marker in lymph node-negative breast cancer patients. *J. Natl. Cancer Inst.*, **85,** 965–970.

SILVESTRINI R. (1994). Cell kinetics: prognostic and therapeutic implications in human tumours. *Cell Prolif.*, **27,** 579–596.

SILVESTRINI R, RAO S, BENINI E, DAIDONE MG AND PILOTTI S. (1995). Immunohistochemical detection of p53 in clinical breast cancers: a look at methodologic approaches. *J. Natl. Cancer Inst.* (in press).

TUNGEKAR MF, GAFFER KC, DUNHILL MS AND MASON DY. (1991). Ki-67 immunostaining and survival in operable lung cancer. *Histopathology*, **19,** 545–550.

VOLM M, DRINGS P, MATTERN J, SONKA J, VOGT-MOYKOPF I AND WAYSS K. (1985). Prognostic significance of DNA patterns and resistance-predictive tests in non-small cell lung carcinoma. *Cancer*, **56,** 1396–1403.